# A Situation-Aware Indoor Localization (SAIL) System Using a LF and RF Hybrid Approach

**DOI:** 10.3390/s18113864

**Published:** 2018-11-10

**Authors:** Jung Kwang Park, Jeeyoung Kim, Soon Ju Kang

**Affiliations:** 1 School of Electronics Engineering, College of IT Engineering, Kyungpook National University, 80 Daehakro, Bukgu, Daegu 702-701, Korea; miff214@naver.com; 2 Center of Self-Organizing Software-Platform, Kyungpook National University, 80 Daehakro, Bukgu, Daegu 702-701, Korea; jeeyoungkim@knu.ac.kr

**Keywords:** WSN, indoor-localization, situation-aware, low-frequency, BLE, RSSI, trajectory tracking, mobile asset management, IoT

## Abstract

Recently, studies focusing on identifying user’s current location for use in a wide variety of differentiated location-based and localization services have steadily increased. In particular, location awareness using wireless communication is gaining attention in indoor environments composed of many obstacles, where GPS signals cannot reach. Previously, studies have focused mostly on location precision, which resulted in using many beacon nodes, not considering the initial installation and maintenance costs, communication robustness, or power consumption. This makes it difficult to apply existing methods to various fields, especially in mobile nodes (i.e., wearable devices, mobile tags, etc.) with limited battery capacity. In this paper, we propose a hybrid situation-aware indoor localization (SAIL) system for real-time indoor localization using a combination of low frequency (LF) and Bluetooth Low Energy (BLE) 4.0. This approach allows us to work with limited battery capacity mobile devices, and identify tagged mobile nodes and their current location in relevance to the anchor node. In our experiment, we attached one anchor node at the entrance to indoor areas such as office or factory settings. Using our hybrid SAIL system, we were able to detect the passing of a mobile node through the entrance and recognize whether the node is entering or exiting the room by calculating the direction of movement as well as the distance from the entrance. This allowed us to distinguish the precise position in an indoor environment with the margin of error being 0.5 m. The signal attenuation due to obstacles is overcome by using LF communication in the 125-kHz band. This approach enables us to reduce the number of initially installed anchor nodes as well as the power consumption of the mobile node. We propose an indoor position recognition system, namely, the hybrid SAIL system, that can be applied to mobile nodes with limited battery capacity by reducing the system complexity and power consumption.

## 1. Introduction

Location awareness technology using wireless communication channels have been proposed as a promising method in the location-based service (LBS) field and lends to a variety of applications and differentiated services based on current location [1]. Mostly, these types of services are provided based on the user’s location information detected in real time. In recent years, services such as home services, large-scale building guidance services, and wealth management services targeting indoor environments have attracted attention, and the need for services to protect the socially weak such as children and elderly population is increasing [2,3]. Traditionally, GPS signal is used in location recognition systems; however, GPS cannot be adopted in the case of indoor locations, due to its limitation of not being able to accurately detect cluttered indoor environments [4,5,6]. To solve this problem, research on methods for recognizing a position in the room has been gaining attention recently.

These attempts have been aimed at efficient and accurate indoor positioning even in indoor environments with many obstacles. Recently, various studies have been conducted to satisfy these scenarios, proposing positioning methods such as Angle of Arrival (AOA), Time Difference of Arrival (TDOA), Received Signal Strength-based, and cell-based methods using a variety of communication technologies such as infrared (IR), ultrasonic, wireless local area network (WLAN), ultra wide band (UWB), and BLE [7,8]. Each of the aforementioned technologies have advantages and disadvantages, but their common goal is aimed at improving the accuracy of location recognition. Efficiency, installation cost, power consumption, etc., which have trade-offs in the process of improving precision, have relatively low priority [7,8]. However, real-world LBS requires consideration of not only the exact location but also the number of mobile nodes it can recognize and the duration for which it can operate with battery constraints.

Many of the current studies did not satisfy the requirements of real-world location recognition services. Typically, to improve accuracy, a method using a number of beacon devices is not suitable for an actual LBS system [9,10]. Several problems arise as the number of devices increases: the system is difficult to install and maintain, leading to a crowded communication environment. If the number of mobile devices to be tracked increases in an environment where there are beacon nodes as well as other wireless communication devices, recognition problems may also occur.

In addition, the conventional localization method requires periodic scanning and arithmetic processing for the mobile node [9,10,11,12]. Smartphone users with sufficiently large battery capacity will be able to use existing indoor location systems even though the power consumption is significant. However, since mobile device such as assets with limited battery power cannot be used with the likeness of smartphones, we noted that there is a problem in that it cannot be adopted in existing localization studies.

In this paper, we propose a system for real-time indoor location recognition using a mixture of LF and BLE which can be adapted even in a mobile device with limited battery capacity. The proposed system, namely the hybrid SAIL system, detects mobile nodes passing through the entrance and recognizes the current situation by calculating the direction of movement and distance from the entrance to identify the precise position in the indoor environment. This system uses a hybrid of LF and BLE, taking in the robust characteristic with high transmittance to obstacles of LF and common use and low-power implementation characteristic of BLE. This can reduce the number of initially installed anchor nodes and reduce the power consumption of the mobile node. In addition, the system can be extended to an entire building with a wireless sensor network (WSN) using adjacent anchor nodes. By presenting a location-aware system that can be used in battery-constrained mobile nodes, our system can be applied to a variety of LBS applications such as mobile asset management and tracking, smart factory systems, smart hospital systems and large-scale building guidance systems.

The remainder of this paper is organized as follows. A brief literature review for studies related to localization using WSN is provided in Section 2. The situation-awareness, localization algorithm and sequence diagram are described in Section 3. A large-scale scenario consisting of a distributed system is shown in Section 4. Section 5 describes the experiments and analyzes the results achieved. Finally, we wrap up by concluding and explaining the direction of future work in Section 6.

## 2. Related Research

In Section 2, we show an overview of several widely used methods of the indoor location-aware system. Furthermore, we describe Received Signal Strength (RSS) Indication (RSSI) based localization solutions, especially LF communication and BLE communication, which are typical methods of RSSI based communication. We discuss the characteristics of each communication method, as well as their pros and cons. We also show examples of each communication through existing related studies. 

There have been numerous efforts to solve the location identification problem of cluttered indoor environments. These technologies include IR, computer vision, WLAN, UWB, Ultra High Frequency (UHF), Radio Frequency Identification (RFID), etc. [7,8]. In recent years, algorithm refinement or additional sensors fusion such as accelerometers or gyroscopes [11,13] have been used to find a more accurate position. Indoor position algorithms are generally classified as: RSSI, TOA, TDOA or AOA [7].

Among them, RSSI-based location awareness systems are widely used due to their relatively low operating cost and easy implementation compared to other technologies. RSSI indicates a measurement of the power present in a received radio signal. Localization performance of RSSI-based techniques are affected by the measurement accuracy of RSSI. LF communication and BLE communication are typical examples of RSSI-based wireless communication technologies. In the case of indoor location recognition systems such as the hybrid SAIL system, many studies have been conducted to improve the stability of RSSI even in difficult environments. To compensate for the advantages and disadvantages of each communication characteristic, additional fusion sensors or mixed-type position sensing studies have attracted a growing interest from the research community [11,13,14,15,16]. 

### 2.1. BLE-Based Localization System

BLE is the most commonly used wireless communication technology in RSSI-based localization technology, due to its low-cost and low-power properties. In addition, it has the advantage of high accessibility because various devices including smartphones support BLE communication. Therefore, BLE is traditionally used in most location-based services such as asset tracking and access control. Examples of indoor positioning research can be found in the form of using the triangulation method [17,18] and the fingerprint mapping method [11,19] by installing beacon devices that provide BLE advertisements.

There is a problem in that the accuracy of the position recognition is very low in a non-line-of-sight (NLoS) situation where an obstacle exists between communication nodes. This is because the permeability to an obstacle is relatively low at an ultra-high-frequency band. Since the received signal’s transmittance is low, the received signal is often reflected by surrounding object and not received directly from the source. Thus, the BLE RSSI value is not only inaccurate but also unpredictable. This is called the multipath propagation phenomenon. As the BLE signals are especially affected by multipath propagation and NLoS conditions, it is difficult to obtain accurate distance estimations using this method in cluttered indoor environments [20]. To solve this problem, location recognition studies using BLE devices additionally use a plurality of devices (more than four devices) in a small room to improve the position ratio [9,10,11,17,18,19]. However, when the number of communication devices increases, mutual interference occurs between wireless communications. This is because BLE uses the 2.4-GHz industrial, scientific and medical equipment (ISM) band, which, in recent years, has been shared with many other wireless communication methods such as WiFi, ANT+, Zigbee, etc., thus causing mutual interference. Therefore, it is unsuitable to improve recognition accuracy of location awareness by adding wireless devices indiscriminately because it makes the wireless communication environment crowded. 

The above problems are generalized problems in BLE based location aware systems. To solve this problem, many studies have considered adding additional sensors or using hybrid algorithms. A hybrid Distance Vector (DV)-Hop Algorithm [16] is a mixture of existing DV algorithm and RSSI-based method. This method can infer the inaccuracy of the average hop distance calculation method of the DV algorithm and reduce the distance measurement error between the mobile node and the anchor node using the RSSI value. Model-Based Localization [18] tracks the position of a moving node by integrating unreliable and noisy BLE observations streaming from multiple locations. In this study, an observation model based on Wasserstein distance interpolation combined with the sequential Monte Carlo (SMC) method is used for tracking noisy Bluetooth-data. Using particle filter based on the proximity of beacons [11] is one of the popular location studies using fusion sensors. This research proposes an indoor location recognition algorithm that uses accelerometer and geomagnetic sensors as well as a floor map-based particle filter that uses the proximity of Bluetooth beacons. When a radio wave from a Bluetooth beacon is blocked by an obstacle, acceleration and a geomagnetic sensor are used to reduce accumulated position errors. 

### 2.2. LF-Based Localization System

RFID is a very interesting technology for indoor positioning because of its low operating cost and low power consumption characters. To achieve high localization accuracy with RFID technology, ways to improve the reliability of RSSI in challenging environments need to be studied. In particular, using low frequency (125 kHz band) of RFID (LF-RFID) shows low power consumption and high accuracy even in the presence of obstacles, which can be used to our advantage. 

LF-RFID can be operated with low power [20] because it acts as a passive device using a wake-up signal to operate only when a specific event occurs. An LF communication system is achieved by resonant inductive coupling between transmitter and receiver coils within a reactive near-field [21] region. Mobile nodes with limited battery capacity usually operate in sleep mode (low-power mode), only to be activated by the wake-up signal whenever the LF receiver detects a sufficiently strong voltage in the receiving coils. In this way, it is possible to operate only when position recognition is needed, which reduces power consumption when not in use. LF signal also makes reliable localization in multipath and NLoS environment. It uses a low frequency in the 125-kHz band, which has a long wavelength and has high permeability to obstacles. This allows the signal to penetrate most obstacles with negligible attenuation. This makes the strength signal more reliable for localization in the presence of obstacles, which in turn, results in a more precise location recognition in an environment composed of many obstacles such as an indoor environment [20]. However, owing to the characteristics of the near-field and magnetic induction methods, it is inevitable to use separate hardware [21]. The LF signal has a very small payload, which can transmit valid data. Thus, even though the position localization can be recognized using only the LF, to transmit the information, a separate wireless communication transmitter is required [20,21].

Multiple studies on several position recognition have been carried out, recognizing the above characteristics. Research of LF-RFID based indoor positioning systems [22] considers an indoor positioning technique based on the LF (125 kHz) Magneto-Inductive (MI) communication and studies the challenges faced by this technique due to ferromagnetic objects. LF MI communication is very well suited for indoor positioning applications since LF magnetic fields are not greatly affected by the environment. Live Synergy [23] is an LF based localization system utilized at a large food court for various services. To solve the existing problem of technologies falling short on metrics such as boundary sharpness, robustness against interference, and obstacle penetration, the authors presented the design and evaluation of a wireless proximity detection platform based on magnetic induction. 

## 3. Concept of the Hybrid SAIL System

### 3.1. Method of the Proposed Hybrid SAIL System

**Figure 1:**
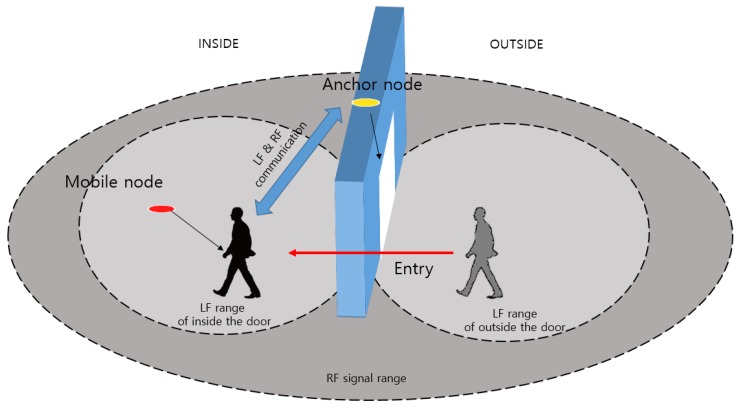
Situation Aware Service Scenario.

Figure 1 shows the system concept of recognizing the indoor position by observing the moving situation of the mobile node. The hybrid SAIL system consists of a mobile node (via wearable device) attached to a user or mobile asset (via mobile tag) and an anchor node attached to an access area. We propose a method to recognize in real time when a user passes through a certain location (anchor node), and to calculate the moving direction and the separation distance from the entrance. 

The system can recognize and monitor the accurate indoor location by using a mixture of the RSSI of LF and BLE signals between the anchor node and mobile node. We used the LF signal of the 125-kHz band for situation recognition. The LF transmitter is designed to be located at the anchor node and the receiver at the mobile node. The anchor node is attached to the door, and two LF antennas are installed inside and outside. The LF range in Figure 1 refers to the range of the LF transmit signal at each antenna. 

We propose hybrid SAIL system that can recognize the precise position using hybrid signal composed of LF and BLE. Basically, it is based on RSSI signal and uses the triangulation method and distance conversion method. The mobile node receives the LF signal and recognizes the access state (entry or exit) and the distance to the entrance by comparing the RSSI values of the received LF signals. The mobile node transmits the recognized information to the anchor node using the beacon signal. 

Unlike the previous information processing method of similar systems, the hybrid SAIL system is composed of distributed computing environments between anchor nodes. An anchor node is connected to other anchor nodes located in nearby rooms through BLE communication to form a distributed network. In this way, only a specific area where a mobile node is located is activated, which can lead to reducing not only the power consumption but also the information throughput. In hybrid SAIL system, anchor node networks with neighboring anchor nodes use BLE communication. Thus, it is possible to synchronize and monitor received information, thereby expanding the recognition range.

### 3.2. Implementation of Hybrid SAIL System

**Figure 2:**
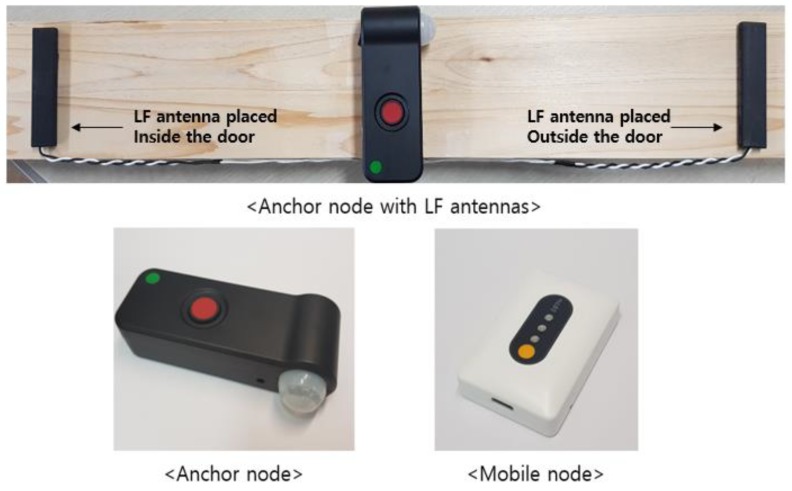
Actual devices of the hybrid SAIL System.

**Figure 3:**
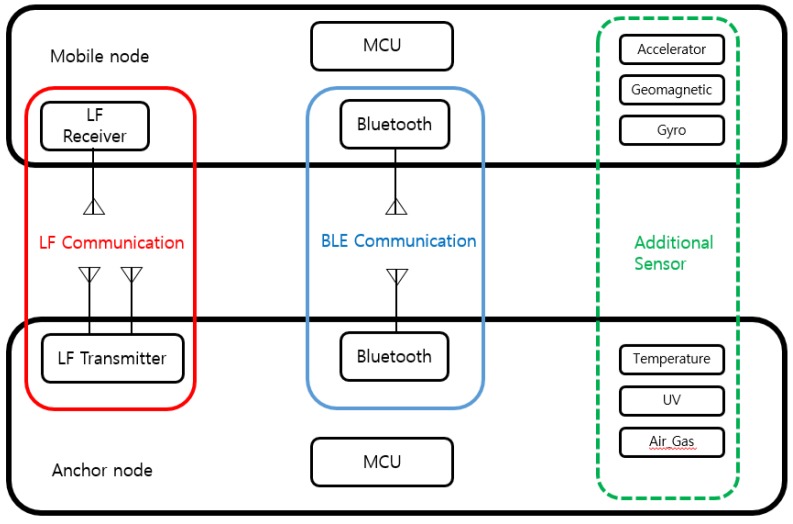
Communication blocks of nodes of the hybrid SAIL System.

Figure 2 shows the hardware prototypes of an anchor node and mobile node, and Figure 3 shows the hardware configuration of the anchor node and mobile node of the proposed indoor location-aware system. These nodes are used for localization of user or mobile property in our experiments. An anchor node is attached to the entrance to which access is desired while keeping information about its current location. Anchor node has two LF antennas which are positioned inside/outside relative to the entrance. Each LF antenna periodically transmits an LF signal in the preamble of the signal about its own position (i.e., whether it is inside or outside the door) and the location information of the attached anchor node. This allows us to determine situation awareness of mobile nodes in real-time by comparing the signals of the two antennas to distinguish whether the mobile node is entering or exiting the room. The size of the anchor node is 121 mm × 60 mm × 32 mm and its total weight is 120 g. A mobile node is worn or attached to a user or mobile property. The mobile node has an LF receiver and receives the LF signal generated from the anchor node. The size of the mobile node is 68 mm × 35 mm × 17 mm including a lithium polymer battery and its total weight is 30 g. Both nodes have BLE communication modules and communicate with each other using Bluetooth. In addition, the anchor node forms a BLE network with adjacent anchor nodes and supports bidirectional communication. Additional sensors may be attached to customize the system for use in different application scenarios. 

### 3.3. Algorithm of Hybrid SAIL System

#### 3.3.1. Situation and Distance Awareness

**Figure 4:**
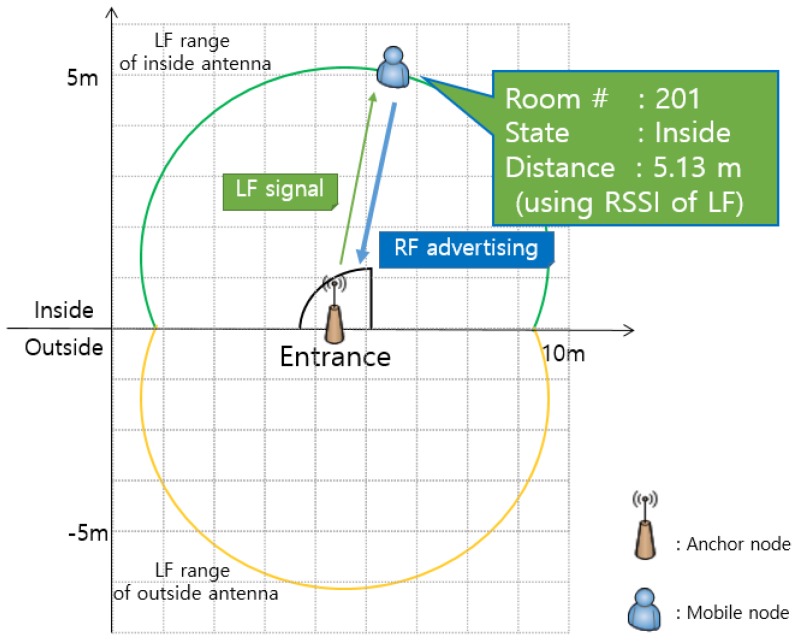
Situation and Distance awareness Between Mobile and Anchor node.

Figure 4 shows that the location information (Room #201, Inside) of the currently located mobile node and the distance (5.13 m) from the entrance can be recognized using the anchor node and two LF antennas installed at the door. 

The mobile node incorporates an LF receiver and receives periodic LF signals transmitted from the anchor node. The LF antennas are installed inside/outside the entrance door. The mobile node can compare the RSSI values of the LF signals transmitted from two antennas and clearly distinguish whether the location is inside or outside relative to the entrance door. That is, it can be determined whether the mobile node has moved in or out. In addition, it is possible to obtain information of the present location. 

The mobile node can obtain the distance between the anchor node and the mobile node through the algorithm of receiving the LF signal and converting the RSSI into distance. That is, with the anchor node attached to the entrance door, the mobile node can determine its distance from the doorway. Since the LF signal has a long wavelength, it is possible to estimate a relatively accurate distance even in a room that contains many obstacles. 

In summary, the mobile node can use the LF signal to determine information about the access area, the entry/exit situation, and the distance from the entrance. However, if only the LF signal is used, it has a limitation in that it cannot recognize the exact position in the room because it knows the distance from the door but does not obtain information about the direction. To solve these limitations, we propose a more precise position recognition method using BLE signals based on situation and distance information. To derive more accurate localization, the mobile node shares the location information by transmitting the obtained information to the anchor node through the BLE advertising signal. 

#### 3.3.2. Accurate Indoor Location Detection

**Figure 5:**
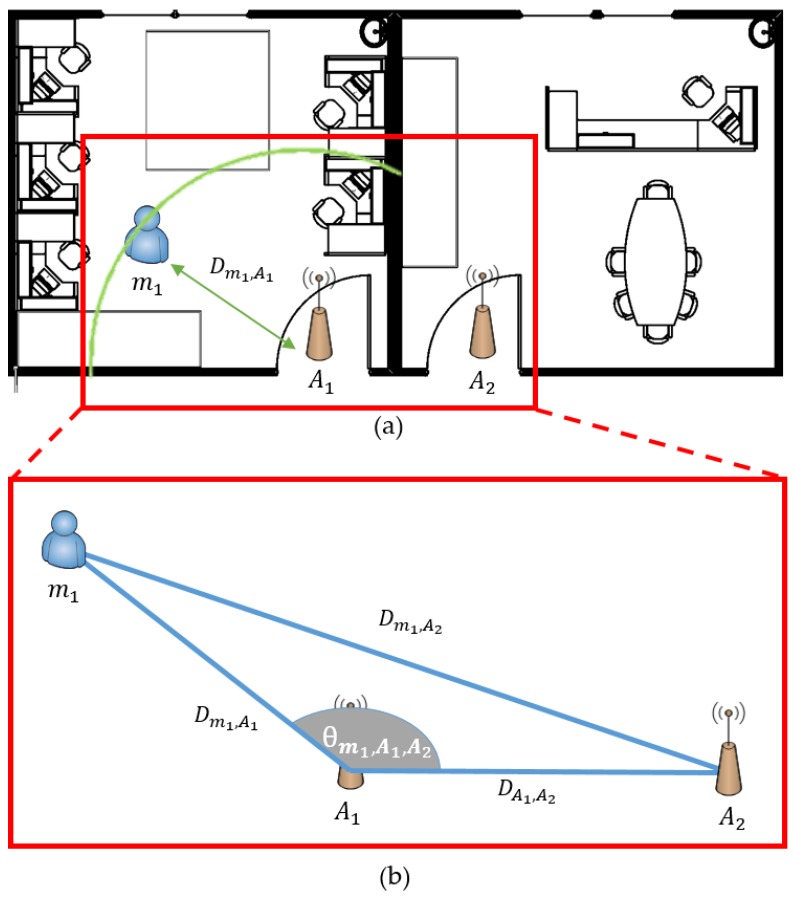
Precise position recognition using RSSI of LF/RF signal: (**a**) distance awareness using LF; and (**b**) angle awareness using BLE.

Figure 5 shows the precise location of the mobile node in an environment with one anchor node at each entrance. The anchor node of the system recognizes the location of the mobile node by constructing the WSN with another anchor node attached to the adjacent entrance. There is an advantage in that it is effective in initial installation cost and maintenance by using a method of attaching only one anchor node to an entrance rather than installing a plurality of devices to the existing systems. 

First, the mobile node advertises its own location, situation, and distance from the door using the LF signal, as shown in Section 3.3.1. The anchor node periodically scans the beacon signal of the neighboring mobile node through the BLE scan mode. Since the beacon signal of the scanned mobile node includes the RSSI value, it is possible to derive the distance between the mobile node and the anchor node. The distance conversion through the RSSI value can be derived from Equation (1):

(1) $$\;\mathrm{Distance}=10^{\frac{Tx\;Power\;-RSSI}{10*N}}\;$$

We want to determine the precise location, which we cannot distinguish by the LF signal alone. We accomplish this by estimating the distance between the mobile and neighboring anchor nodes through the *RSSI* distance transform equation. The *Tx* power of Equation (1) means transmitted power of BLE antenna and we use 4 dBm. RSSI refers to the strength of the received signal when an anchor node receives the BLE advertising signal from the mobile node. *N* is the correction constant, which we set as 2. 

(2) $$\;D_{source,\;\;\;destination}\;$$

$$\;m_{i}:\;mobile\;node,\;i=1,2,\ldots ,n\;A_{i}:\;anchor\;node,\;i=1,2,\ldots ,n\;D_{m_{1},A_{1}\;}:\;mobile\;node\;1\;to\;anchor\;node\;1^{\prime }s\;distance\;D_{A_{1},A_{2}\;}:\;anchor\;node\;1\;to\;2^{\prime }s\;distance\;$$

***D*** is the inter-device distance of the proposed system, which is the distance from the source to the destination based on the subscripts ***m*** being the mobile node and ***A*** being the anchor node attached to each door. Since a plurality of nodes are used, the applicable device is referred to using the number (i=1,2,…,n). For example, $D_{m_{1},A_{1}\;}$is the distance between $m_{1}$ and $A_{1}$, and $D_{A_{1},A_{2}}$ is the distance between $A_{1}$ and $A_{2}$. Since the distance information between the mobile node and all anchor nodes can be determined, a more precise position can be recognized indoors by using the following second law of cosines:

(3) $$\;\theta_{m_{1},A_{1},A_{2}}=\cos^{-1}\frac{D_{m_{1},A_{1}\;}{}^{2}+D_{m_{1},A_{2}}{}^{2}-D_{A_{1},A_{2}}{}^{2}}{2D_{m_{1},A_{1}\;}D_{m_{1},A_{2}}}\;$$

$\theta_{m_{1},A_{1},A_{2}}\;$means that the angle of $m_{1}$ is away from $A_{1}$ and $A_{2}$. In Section 3.3.1, information was obtained about the current entrance and distance information from the door. However, in this section, to solve the issue of when the information about direction is not obtained, BLE communication is established with the anchor nodes installed in the surrounding doorway, and the above induction formula is used. By adding θ information derived from the formula based on the distance from the entrance to the mobile node, the mobile node can acquire all the information about the distance and the current direction and can determine the accurate indoor location in real-time.

### 3.4. Sequence Diagram

**Figure 6:**
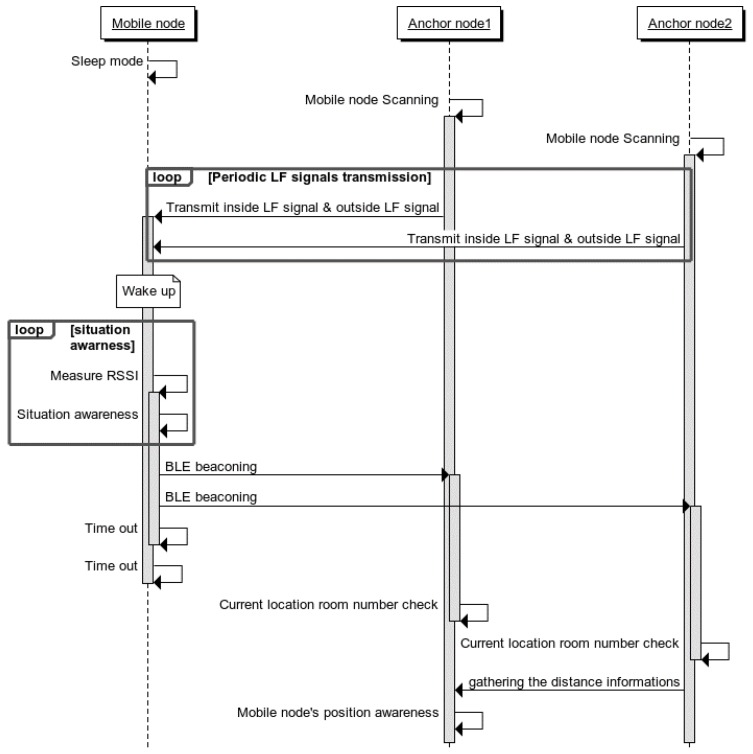
Situation-aware indoor localization system service sequence diagram.

Figure 6 shows the sequence of steps to identify the indoor position between the devices (mobile node and anchor node) of the proposed system. First, a plurality of anchor nodes is installed at the entrance of each area where the indoor location is to be recognized.

The anchor node periodically transmits the LF signal indicating the inside and outside of the entrance through two LF antennas. The mobile node attached to the moving object collects all LF signals generated from the anchor node through the LF receiver. When the received LF signal is present, information of the zone where the LF signal is currently located can be known by using the strongest RSSI value based on the LF signal value. In addition, since the inside and outside information can be obtained, the mobile node can recognize its current situation of entry or exit.

After obtaining the information of the zone where the mobile node has entered, the RSSI value of the LF signal received from the anchor node installed in the corresponding area is applied to the distance estimation algorithm. This allows the mobile node to determine its distance from the door. To share the location recognized by the mobile node, the mobile node advertises the BLE beacon signal with information on the area where the mobile node is located. Not only the anchor node in the access area but also the adjacent anchor nodes receive the beacon signal of the mobile node. 

In this process, the position of the mobile node becomes more precise. Each anchor node receives an RSSI value when it receives a BLE beacon signal. The distance between the mobile node and the neighboring anchor node can be derived through Equation (1). The neighboring anchor nodes transmit the derived internode distance information to the anchor nodes of the entrance area again. The anchor node of the entry area collects all distance information between the mobile node and the anchor nodes so that the distance and direction of the mobile node can be derived by applying the algorithm used in Equation (3). 

## 4. Concept of Self-Organizing Dynamic Space Configuration

### 4.1. Configuring the Overlay Network 

**Figure 7:**
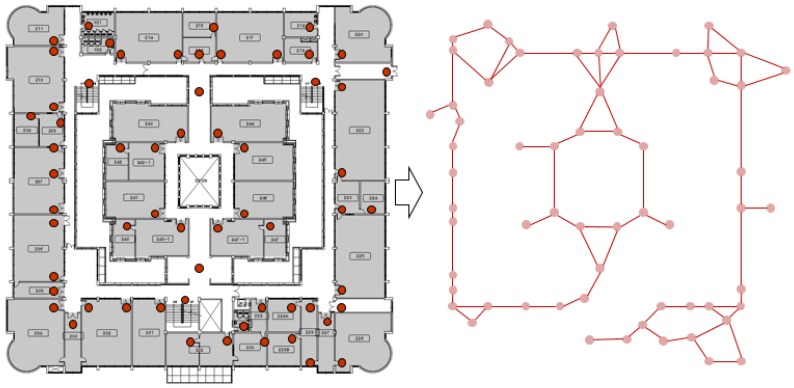
Configuration of the overlay network.

Figure 7 shows what the configuration of the proposed system would look like if scaled up in a real physical environment. Due to the distributed, decentralized nature of the hybrid SAIL system, the performance of our suggested platform is minimally affected even when the number of nodes increase. We also show what the network configuration would look like between the anchor nodes that are installed at the entrance of each room in a large area consisting of >30 rooms. The anchor nodes are aware of the location information about the room where they are installed and use BLE communication to search for and connect to the adjacent anchor nodes to form a BLE connection. One anchor node is composed of a scatter-net network with two or more nodes using BLE communication. 

By adopting this scheme, the networks of adjacent anchor nodes are continuously connected, and the entire network configuration is largely connected to one network in a relay protocol. Since all networks are interconnected, we can increase the data transmission and processing speed of the proposed system. In addition, if an anchor node has a problem and fails, this can be detected in real time, which is advantageous for maintenance. Combining the characteristics of our distributed hybrid SAIL system and the network topology of anchor node network, we show the scalability of our system. Actual implementation of this larger scenario and high-level applications such as navigation services will be explored in our future work.

### 4.2. Distributed System Configuration

**Figure 8:**
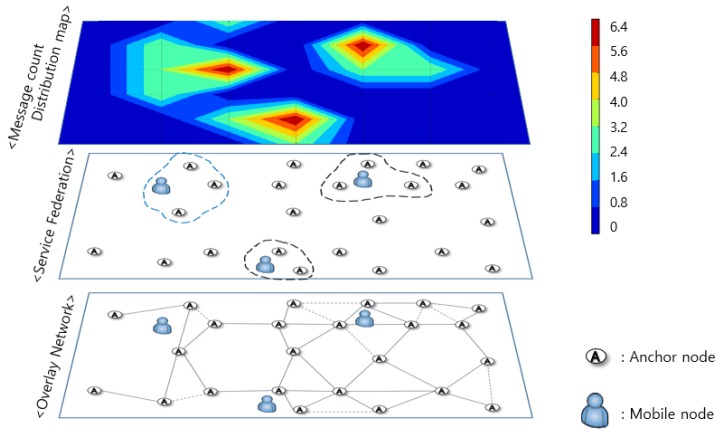
Distributed system configuration between anchor nodes.

Figure 8 shows a simulation of the operation mechanism and the amount of BLE communication information generated when multiple mobile nodes exist in the entire system. The system consists of a distributed computing system, which processes data directly from each department where the data are generated, and not in a large centralized system. That is, data transmission occurs only in the indoor area where the mobile node is located so that only the anchor node adjacent to the mobile node transmits and processes the data. Anchor nodes in areas where the mobile node is not present do not need to send messages, which reduce power consumption as well as the message transmission load for the entire system. This allows us to provide a system using lower power and low data overhead. This is also the reason that our system can scale up with minimal performance loss. 

## 5. Performance Evaluation

### 5.1. Test Environment

To evaluate the performance of the hybrid SAIL system, three different localization tests (Experiments 1–3) were carried out. This section describes the environment, equipment used, setup, and procedures used during these experiments.

For the experiments, the hybrid SAIL system was tested in an environment that includes NLoS conditions, with the presence of various obstacles, both metallic and not and humans. The testing environment, which consisted of indoor office spaces with a total area of around 65${\;m}^{2}$, contained inner walls forming two rooms, as shown in Figure 10a. The floor-to-ceiling height was approximately 3 m. In office environments, there are metallic machines and tools present. In addition, this environment contains wooden and metallic cabinets, bookshelves, partitions, window frames and so on. The building itself is a typical concrete office building with the floors, ceilings and walls containing metallic beams and steel bars.

Experiment 1 evaluated how well the moving mobile node can detect its current situation. This experiment examined the entry/exit situation with a 1-m-wide door. Two LF antennas were installed inside and outside at 50 cm apart from the entrance door. The LF signal intensity was analyzed by moving about 12${\;m}^{2}$ based on the entrance door. In Experiment 2, the anchor node was attached to two doors in the environment of Figure 10a, and the measurement error of the actual position was analyzed by recognizing the positions of the nine mobile nodes in real time. Experiment 3 evaluated how quickly the location can be recognized when the mobile nodes increase. We increased the number of mobile nodes from 5 to 20 by increments of 5 and measured the time in milliseconds until the location confirmation was completed for all mobile nodes. 

### 5.2. Results and Analysis

**Figure 9:**
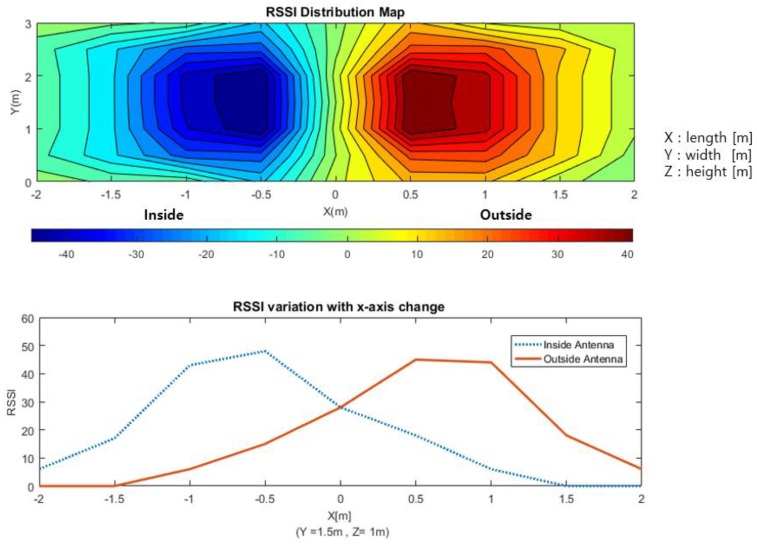
Comparison of LF signals for situation-awareness. (Experiment 1).

Figure 9 shows the RSSI values received from two LF antennas installed inside and outside the anchor node of the entrance. In Figure 9, the x-axis of the graph represents the length, y-axis indicates the width and z-axis indicates height. The LF signal strength was measured every 0.5 m by keeping the 1 m height of z-axis and moving along the x and y-axis in an 4 m×3 m×3 m indoor space. The blue graph shows the LF antenna value inside the door, and the red graph shows the LF antenna value outside the door. To compare RSSI values received from two different LF antennas, we plotted the graph by assigning a negative sign to the inside antenna signal. The signals of the two LF antennas show a difference in signal strength value at the same coordinate point when moving left and right based on the 0-m point of x-axis where the door is located. That is, on the x-axis at 0 m or more, the value of the inside LF antenna is received more strongly, and it can be accurately judged that the mobile node is currently entering through the entrance. The exit situation is also recognizable in the same way. It is confirmed that it is possible to accurately determine the entry and exit situation of the mobile node through the LF antennas installed inside and outside of the entrance. 

**Figure 10:**
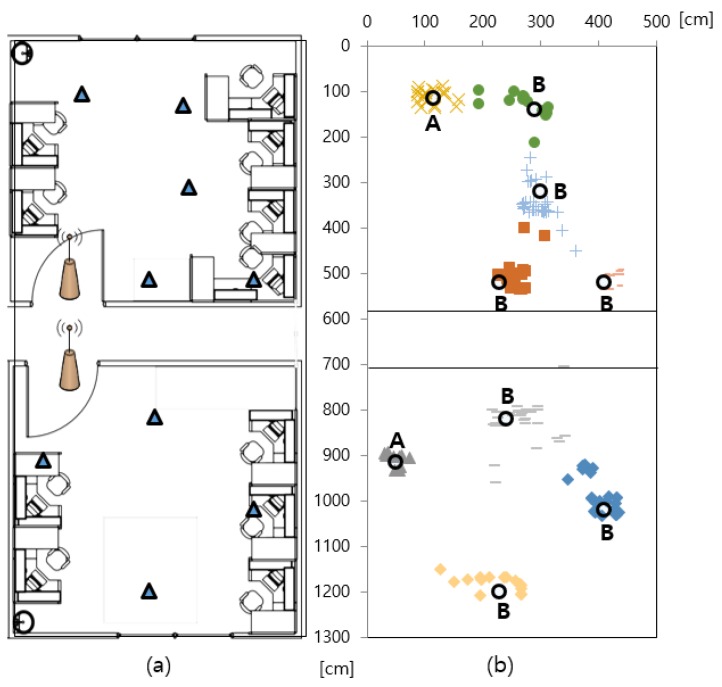
Comparison of measurement positions using proposed system: (**a**) test bed environments; and (**b**) localization results (Experiment 2).

Table 1 lists the specific margin of estimation errors for the indoor positions of the mobile node using the hybrid SAIL system. Table 1 shows the number of mobile nodes in ascending order based on the x-axis in Figure 10b. If it is in line-of-sight (LoS) condition in which there is no communication obstacle between the anchor node and the mobile node, the accuracy of the position recognition is 30 cm or less because the signal strength is relatively clear. This kind of position is marked as “A”. However, if it is in NLoS condition, in which there is a communication obstacle such as walls and cabinets not shown in the picture, the signal intensity value changes owing to the reflection of the radio wave, and a recognition error of 50 cm at maximum can be confirmed. This kind of position is marked as “B”. 

In conclusion, we have shown that the position recognition of the proposed method has an accuracy of less than 50 cm in terms of average margin of error regardless of the position and obstacles. The hybrid SAIL system proposed in this study shows that accurate indoor location is possible using one node, while existing indoor location awareness research uses at least 4–8 beacon nodes. Thus, we claim that it is possible to obtain a relatively accurate indoor mobile node position while greatly reducing the initial installation cost. 

**Figure 11:**
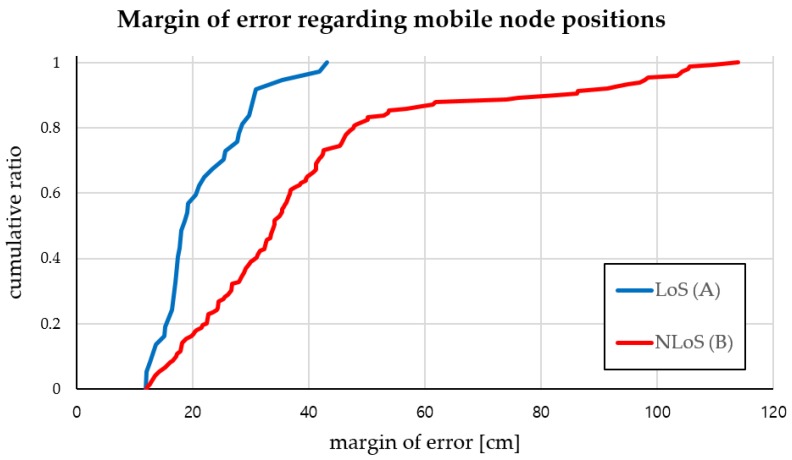
Margin of error regarding actual mobile node positions shown in both LoS and NLoS conditions.

Cumulative ratio for margin of errors of the mobile node positions for two different conditions are shown in Figure 11. Blue graph in LoS condition keeps positioning error below 18 cm for 50% and below 30 cm for 90% of the test points while red graph in NLoS condition achieves positioning error below 33 cm for 50% and below 60 cm for 90% of the tasks. Although the error is increasing in the NLoS situation, the maximum average value per position is still less than 50 cm. The hybrid SAIL system we proposed based on LF and RF has great potential to reduce initially installed anchor nodes and to become a low cost, power efficient solution in cluttered indoor environments. 

**Figure 12:**
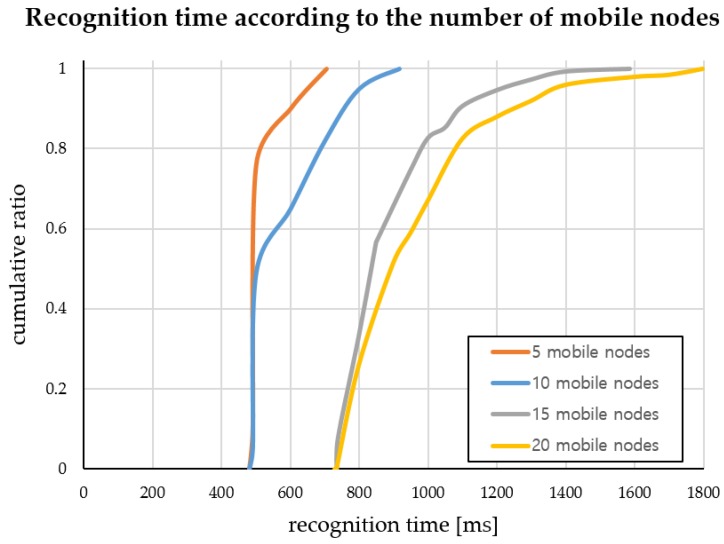
Recognition time according to the number of mobile nodes increments of 5 (Experiment 3).

We implemented a mobile node localization service using the hybrid SAIL system in Figure 10. To prove the scalability and stability of the system, we performed Experiment 3 to measure the recognition time by increasing the number of mobile nodes. This experiment was performed to test the concentration of mobile nodes at one anchor node in an environment where there are four anchor nodes. The recognition time measurement was taken repeatedly for 50 times for each set of mobile nodes. Figure 12 shows the cumulative graph of the recognition time taken to recognize all mobile nodes by increasing the number of nodes from 5 to 20 by increments of 5, measured in milliseconds. The average recognition time is provided in Table 2. 

We have confirmed that the time taken to recognize the location of the 20 mobile nodes is within 1 s. Although the total recognition time increases gradually as the number of mobile nodes increases, it is possible to recognize the location of all mobile nodes in a relatively short time even in a concentrated situation of one anchor node and to provide stable service. 

## 6. Conclusions

In this paper, we proposed the hybrid SAIL system, a situation aware indoor localization system which uses a combination of LF and BLE signals to locate mobile nodes and their current situation (entry/exit). LF is used to effectively measure the distance from the anchor node, while BLE is used to determine the direction of the mobile node. Unlike previous studies that advertised simple information in anchor nodes and calculated precise locations in mobile nodes, this study changed the method in which a mobile node transmits a simple advertisement and the anchor node calculates the precise position, leading to effective application of mobile nodes with limited battery capacity while considering efficiency, installation cost, and power consumption as well as the accuracy of indoor location awareness. In addition, as a distributed computing system, the system scalability is increased. Eliminating unnecessary information throughput helps reduce the system complexity as well.

Through performance evaluation, we established that the situation of entry/exit of a mobile node can be determined in real-time and that we can also accurately recognize the direction of travel as well as the indoor location of the mobile node. We also confirmed that it is possible to recognize similarly precise positions (maximum average localization error value per position <50 cm at most), even though we reduced the number of nodes by installing one anchor node per entrance. We also show that, even when many mobile nodes congest a given area, the time it takes to determine user location only diminishes minutely (average time for 5 nodes and 20 nodes difference is less than 0.5 s).

Our proposed system can be applied to a wide variety of LBS applications such as mobile asset management, smart factories and patient monitoring systems since it is more applicable by reducing the power consumption for mobile nodes as well as the number of anchor nodes. In the future, we will examine the robustness of the system by increasing the number of experimental mobile nodes. We also plan to conduct experiments that cover larger areas, such as an entire building as well as high-level application scenarios such as navigation services.

## Figures and Tables

**Table 1:** Accuracy of localization at each position.

Mobile Node Number	x Coordinates (cm)	y Coordinates (cm)	Average Localization Error of Proposed System (cm)
1	50	915	19
2	115	115	24
3	230	520	43
4	230	1200	47
5	240	820	48
6	290	140	45
7	300	320	45
8	410	520	27
9	410	1020	43

**Table 2:** Time of mobile nodes recognition.

Number of Mobile Nodes	Max Time (ms)	Min Time (ms)	Average Time (ms)
5	704	488	522
10	916	488	579
15	1585	736	884
20	1797	736	953
